# A biorefinery scheme to fractionate bamboo into high-grade dissolving pulp and ethanol

**DOI:** 10.1186/s13068-017-0723-2

**Published:** 2017-02-10

**Authors:** Zhaoyang Yuan, Yangbing Wen, Nuwan Sella Kapu, Rodger Beatson, D. Mark Martinez

**Affiliations:** 1 Department of Chemical and Biological Engineering, University of British Columbia, 2360 East Mall, Vancouver, BC V6T 1Z4 Canada; 2 Tianjin Key Laboratory of Pulp & Paper, Tianjin University of Science and Technology, Tianjin, 300457 China; 3 Chemical and Environmental Technology, British Columbia Institute of Technology, 3700 Willingdon Ave, Burnaby, V5G 3H2 Canada

**Keywords:** Bamboo, Alkaline pretreatment, Dissolving pulp, Bioethanol, Silica, Lignin

## Abstract

**Background:**

Bamboo is a highly abundant source of biomass which is underutilized despite having a chemical composition and fiber structure similar as wood. The main challenge for the industrial processing of bamboo is the high level of silica, which forms water-insoluble precipitates negetively affecting the process systems. A cost-competitive and eco-friendly scheme for the production of high-purity dissolving grade pulp from bamboo not only requires a process for silica removal, but also needs to fully utilize all of the materials dissolved in the process which includes lignin, and cellulosic and hemicellulosic sugars as well as the silica. Many investigations have been carried out to resolve the silica issue, but none of them has led to a commercial process. In this work, alkaline pretreatment of bamboo was conducted to extract silica prior to pulping process. The silica-free substrate was used to produce high-grade dissolving pulp. The dissolved silica, lignin, hemicellulosic sugars, and degraded cellulose in the spent liquors obtained from alkaline pretreatment and pulping process were recovered for providing high-value bio-based chemicals and fuel.

**Results:**

An integrated process which combines dissolving pulp production with the recovery of excellent sustainable biofuel and biochemical feedstocks is presented in this work. Pretreatment at 95 °C with 12% NaOH charge for 150 min extracted all the silica and about 30% of the hemicellulose from bamboo. After kraft pulping, xylanase treatment and cold caustic extraction, pulp with hemicellulose content of about 3.5% was obtained. This pulp, after bleaching, provided a cellulose acetate grade dissolving pulp with α-cellulose content higher than 97% and hemicellulose content less than 2%. The amount of silica and lignin that could be recovered from the process corresponded to 95 and 77.86% of the two components in the original chips, respectively. Enzymatic hydrolysis and fermentation of the concentrated and detoxified sugar mixture liquor showed that an ethanol recovery of 0.46 g/g sugar was achieved with 93.2% of hydrolyzed sugars being consumed. A mass balance of the overall process showed that 76.59 g of solids was recovered from 100 g (o.d.) of green bamboo.

**Conclusions:**

The present work proposes an integrated biorefinery process that contains alkaline pre-extraction, kraft pulping, enzyme treatment and cold caustic extraction for the production of high-grade dissolving pulp and recovery of silica, lignin, and hemicellulose from bamboo. This process could alleviate the silica-associated challenges and provide feedstocks for bio-based products, thereby allowing the improvement and expansion of bamboo utilization in industrial processes.

## Background

Concerns over climate change have driven the development of a bio-based economy in which energy and numerous consumer products are manufactured from the renewable lignocellulosic feedstocks. Bamboo, a fast-growing species that becomes harvest-ready in 3–5 years and has comparable contents of cellulose and hemicelluloses to those of hardwoods and softwoods, is an attractive feedstock in the proposed biorefinery concept [1–3]. Bamboo is a highly abundant natural resource worldwide [4]. In China alone, there are approximately 300 species in 44 genera, occupying 33,000 km^2^ of the country’s total forest area [5]. Indeed, there are difficulties to utilize bamboo in traditional pulping and biorefinery processes. Compared to wood, bamboo contains a much higher level of silica [6–8]. Silica creates downstream problems during pulping and bioconversion processes, and even causes complications in the effluent streams [9–14]. Therefore, removing silica from raw materials prior to subsequent processing steps is of great importance in expanding bamboo usage.

Alkali can be used to dissolve silica and transfer the soluble silicates into the bulk liquor; this provides a means of extracting silica prior to subsequent processing. Our earlier studies have shown that most of silica in bamboo could be pre-extracted with sodium hydroxide (NaOH) and recovered as by-products by lowering liquor pH [15]. The recovered silica can be used to produce nanosilica particles, composite fillers, pharmaceuticals, and catalysts [16, 17]. The hemicellulose extracted during the alkaline pretreatment can be used for the generation of various products such as bioethanol, furfural, acetone, or papermaking additives [18–20]. The silica-free cellulose-rich substrate can be an excellent resource for bio-based products such as biofuels, biochemicals, and dissolving pulp.

Dissolving pulp (i.e., pulp with >92 wt/wt % α-cellulose), a specialty starting material from lignocellulosic biomass, is of growing interest. It is used for the production of viscose rayon, cellulose acetate, and nanocrystalline cellulose (NCC), carboxymethyl cellulose (CMC), cellulose nitrate, paints, and liquid crystal displays [2, 21, 22]. Alkaline pretreatment can improve delignification and hence reduce the alkali charge required in subsequent cooking step [15, 23–25]. However, alkaline treatment step is not as effective as acidic pre-hydrolysis in hemicellulose removal [23, 25, 26]. Therefore, to produce high-grade dissolving pulp from bamboo, it is necessary to incorporate a hemicellulose removal step after kraft pulping. Among the investigated methods, xylanase treatment and cold caustic extraction (CCE) are considered viable options in the removal of hemicellulose after kraft pulping of hardwood and several non-wood pulps [22, 27–29]. However, limited studies have been published on the removal of hemicellulose from bamboo kraft pulp by treatments with xylanase or CCE [30].

The alkaline pre-extraction of silica not only eliminates silica in the pulp, but also enables the recovery of relatively clean lignin with low ash content from the black liquor generated during kraft pulping. Lignin is also a sustainable resource for various lignin-derived products [31]. After separating lignin, dissolved hemicellulosic and cellulosic sugars in the black liquor can be separated and recovered by nanofiltration [32]. Sugars obtained from alkaline pre-extraction liquor and black liquor can be hydrolyzed and fermented to produce ethanol. The residual sodium-rich liquor will be sent to the chemical recovery cycle for the recovery of the spent inorganic chemicals (NaOH and Na_2_S).

In the work described in this paper, an integrated biorefinery scheme that can produce high-purity dissolving pulp, bioethanol, and recover high-purity silica and lignin is configured based on the traditional kraft pulping process. Four alkali reagents, sodium hydroxide (NaOH), potassium hydroxide (KOH), ammonia (NH_4_OH), and calcium hydroxide (Ca(OH)_2_), were investigated for the removal of silica and hemicellulose from bamboo. The alkaline-pretreated chips were subjected to kraft pulping, xylanase treatment, cold caustic extraction (CCE), and bleaching stages to produce dissolving pulp. The recovered sugars from alkaline pretreatment liquor and black liquor were mixed and enzymatically hydrolyzed to produce a monomeric sugar hydrolysate. The sugar hydrolysate was fermented using an engineered strain, *Saccharomyces cerevisiae* BSIF, for the production of ethanol. A mass balance of the overall process was established to evaluate the feasibility of this proposed scheme.

## Experimental

### Raw materials

Bamboo chips, prepared from 3- to 7-year old trees, were provided by the Lee & Man Paper Manufacturing Ltd. China. The obtained chips were washed with deionized water at a liquid-to-wood ratio of 20 L/kg using a laboratory mixer to remove impurities, such as soil and sand. The washed chips were air-dried for approximately for 24 h and then stored at 4 °C until used for subsequent experiments. The moisture content of the bamboo chips was about 22%. Compositional analysis of the raw bamboo chips shows that it contains 47.3% glucan, 21.8% hemicellulose (20.3% xylan, 0.7% galactan, 0.8% arabinan, mannan undetected), 25.3% lignin (sum of acid-soluble and insoluble lignin), and 1.12% silica on a dry basis.

A commercial xylanase was provided by Novozymes (Tianjin, China). The recommended conditions for this xylanase were temperature (40–80 °C) and pH value (5–8). The highest activity (8997 IU/g) occurred at 60 °C and pH 6. Commercial cellulase and hemicellulase, named Cellic CTec2 and HTec2, respectively, were provided by Novozymes Investment Co. Ltd (Tianjin China). The enzymatic activities of Cellic CTec2 and HTec2 were 113 FPU/mL and 2.94 IU/mL, respectively. All other chemicals used in this study were reagent grade and purchased from Fisher Scientific, Canada.

### Alkaline pretreatment of bamboo chips

Four alkali reagents were investigated in this work. Alkaline pretreatment experiments were carried out at 95 °C and alkali charge of 3.0 mmol/g oven-dried (o.d.) bamboo biomass for 150 min using a rotating reactor system (Aurora Products, Savona, BC, Canada) which consists of 4 stainless steel digesters of 2 L each placed in a single rotating frame. Reactors were routinely rotated at 50 rpm with 60-s clockwise rotations followed by 60-s counter clockwise rotations throughout the reaction process. The liquid-to-wood ratio was fixed at 4 L/kg. For the alkaline pretreatment run, bamboo chips of 100 g oven dried (o.d.) and the calculated volume of distilled water and alkali solution were mixed and placed in a digester. Subsequently, the reactor was placed in the digester system for alkaline pretreatment. The temperature ramp-up time was kept constant at 25 min. Time zero (for pretreatment) was taken to be the time the reactor reached the target temperature. Upon completion of a run, the vessel was rapidly cooled in an ice–water bath and the treated chips were recovered from the liquor through filtration. The chips were washed with distilled water and stored at 4 °C for further use. The alkaline pre-extraction liquor was collected and stored at 4 °C until used for experimentation. All experiments were performed in duplicate.

### Dissolving pulp production

#### Kraft pulping

Pulping of pre-extracted bamboo chips was conducted with the same reactor system used for alkaline pretreatment. The charge of effective alkali (EA, calculated as Na_2_O) on bamboo chips (o.d.) ranged from 18 to 22% and the sulfidity (percentage of sodium sulfide expressed as Na_2_O) ranged from 0 to 40%. Two temperatures, 160 and 170 °C, were investigated. The liquid-to-wood ratio, heat-up time, and cooking time at the maximum temperature were fixed at 4 L/kg, 75 and 80 min, respectively. For each cook, 100 g o.d. extracted bamboo chips and the calculated volume cooking chemicals and deionized water were placed in the reactor and mixed for 10 min. Afterwards, the cooking process was carried out according to the condition being investigated. After cooking, the reactor was rapidly cooled and kraft pulp was recovered using filtration. The kraft pulp was thoroughly washed with distilled water until the pH of the filtrate reached neutral. The total pulp yield was determined. Then the pulp was disintegrated and screened using a vibrating flat screen with 0.15-mm-wide slots. The accepted pulp was collected and homogenized to determine the screened yield and rejects content. The screened pulps were put in polyethylene bags and stored at 4 °C for further measurement and xylanase treatment.

#### Xylanase treatment of kraft pulp

A 20 g (o.d.) of kraft pulp was treated with xylanase (4, 8, and 16 U/g o.d. pulp) at 10% consistency for 1–12 h at 60 °C in phosphate buffer (pH 6) using a polyethylene bag. The samples were kneaded in a 30-min interval. After completion of the xylanase treatment, the enzymes were denatured by boiling the samples in water for 15 min. Then, the pulps were collected through filtration and washed with 1000 mL deionized water. The treated pulp samples were stored at 4 °C for subsequent analyses. Experiments were performed in triplicate.

#### Cold caustic extraction (CCE) of kraft pulp

The CCE experiments were carried out in a laboratory water bath. A 20 g (o.d.) pulp was treated with NaOH concentrations (4–12%) at 30 °C for two times (45 min) at pulp consistency of 10%.

The sequential treatment of enzymatic and CCE of the kraft pulp was investigated as described above.

#### Pulp bleaching

The sequential treated pulp was bleached to full brightness with a D-E-D sequence, in which D is chlorine dioxide and E is an alkaline extraction. According to our earlier study [30], Table 1 shows the conditions used for each bleaching stage.

**Table 1:** The D-E-D bleaching conditions

Conditions	D1	E	D2
Consistency (%)	10	10	10
Temperature (°C)	70	80	80
Time (min)	90	60	160
ClO_2_ as Cl_2_ (% of dry weight pulp)	1.5	–	0.5
NaOH (% of dry weight pulp)	–	1.2	–
Final pH	2.2	10.8	4.2

### Recovery and utilization of dissolved materials in the spent liquors

#### Separation of silica, lignin, and sugars from the liquor

According to our preliminary study on precipitating silica from the alkaline pre-extraction liquor (APEL), silica in the APEL was separated by reducing the pH of the liquor to pH 8 by bubbling carbon dioxide (CO_2_) at 60 °C. After silica separation, the hemicellulosic sugars were filtered using a cellulose-acetate-based nanofiltration membrane (GE Osmosis) with a high-pressure stirred cell (Sterlitech HP4750, USA) at pressure of 500 Psi for 150 min.

Lignin dissolved in the black liquor obtained from kraft cooking was isolated by reducing the pH of the black liquor to pH 2 with 72% sulfuric acid (H_2_SO_4_). The precipitate was collected through filtration. After lignin separation, the sugars in the black liquor were concentrated by membrane filtration as described above to recover carbohydrates.

The silica and lignin precipitates were air-dried overnight and vacuum-dried at 45 °C for 48 h to obtain constant weight. The sugars concentrated from the APEL and black liquor were mixed in conical flask with a laboratory mixer at 150 rpm for 30 min and stored at 4 °C for further analysis and experimentation.

#### Detoxification of recovered sugars

Overliming was selected as detoxification procedure to remove inhibitory compounds, such as phenolic compounds, from the sugar retentate mixture following Martinez et al. [65]. Briefly, Ca(OH)_2_ solution was added to the liquor until pH 10. Then, the liquor was agitated (250 rpm) in an orbital shaker at 50 °C for 30 min. Finally, it was centrifuged at 3500 rpm from 10 min for solid separation (BT5, Hunan Labwe Scientific, China). The obtained liquid was used for enzymatic hydrolysis and fermentation.

#### Enzymatic hydrolysis of sugars

The resulting supernatant was adjusted to pH 5.0 prior to enzymatic hydrolysis with 1 mol/L H_2_SO_4_ or NaOH. The enzymes used for hydrolysis, Cellic CTec2 and HTec2, were present in a mass ratio of 2:3, respectively, based on the protein content measured according to Bradford assay (Sigma-Aldrich, St. Louis, MO, USA). The total charge of Cellic CTec2 plus HTec2 was 1–10 mg total protein content per g sugar in the recovered liquid. The hydrolysis was carried out at 50 °C and pH 5.0 for 72 h. Samples were periodically taken for sugar analysis using HPLC. After hydrolysis, the reducing sugar concentration was adjusted to 60 g/L by evaporation or addition of water. The obtained hydrolysate was stored at −20 °C prior to HPLC analysis and fermentation. One control experiment without using enzymes was carried out. All the experiments were performed in triplicate.

#### Fermentation

The xylose-fermenting *S. cerevisiae* BSIF was cultured in medium containing glucose (20 g/L), yeast extract (10 g/L), and peptone (20 g/L) [33]. The yeast was aerobically grown at 30 °C for 2 days with orbital shaking at 200 rpm. Then pre-cultured cells were harvested by centrifugation (BT5, Hunan Labwe Scientific, China) to prepare *S. cerevisiae* cell concentration of 50 g/L. Ethanol fermentations were conducted under an anaerobic condition. For the fermentation experiments, 125-mL serum bottles were employed. For one fermentation experiment, 50 mL of overliming-detoxified and enzymatic hydrolyzed sugars solution was added into the serum bottle. To initiate fermentation, the initial yeast cell concentration was 1.0 g/L. The fermentation was carried out at 30 °C with orbital shaking at 150 rpm and pH 6.0. The ethanol yield to consumed sugar (g/g) was calculated as the ratio of ethanol to consumed sugar amount. The efficiency of sugar conversion to ethanol (%) has been estimated by the ratio of ethanol yield to the theoretical value of ethanol yield (0.51 g/g of sugar). All fermentations were performed in triplicate.

### Analytical determinations

The moisture content of pulp was measured by drying at 105 ± 2 °C to constant weight. The chemical composition of the bamboo chips and pulps were analyzed following National Renewable Energy Laboratory (NREL) standard protocols [34]. Briefly, the chips were air-dried and ground to pass through 40 mesh using a Wiley mill. The powdered samples were then digested by a two-step H_2_SO_4_ hydrolysis protocol. For polysaccharide analysis, acid hydrolysates (liquid samples) were recovered by filtration through medium porosity filtering crucibles (Fisher Scientific Co., ON, Canada), and an internal standard fucose was added. These samples were re-filtered using 0.2-μm syringe filters (Chromatographic Specialties, Inc. ON, Canada) for HPLC. A Dionex ICS 5000+ HPLC system fitted with an AS-AP autosampler was used to separate the monomeric sugars in the samples at 45 °C, against sugar standards, on a Dionex CarboPac SA10 analytical column. 1 mM NaOH at 1 mL/min flow was the mobile phase, and the sugars were quantified using electrochemical detection and Chromeleon software (Thermo Fisher Scientific, MA, USA). High-purity monomeric sugar standards, arabinose, galactose, glucose, xylose, and mannose, were purchased from Sigma-Aldrich (ON, Canada).

Acid soluble lignin in the two-step hydrolysate was measured at wavelength 205 nm using a UV–Visible spectrophotometer [35]. Acid insoluble lignin was determined gravimetrically according to Sluiter et al. [34]. The ash content of bamboo chips and pulp was determined according to TAPPI T211 om-02. Detailed analysis of the metal composition of ash was done using inductively coupled plasma time of light mass spectrometry (iCP-TOFMS) [36]. The α- and β-cellulose contents of bamboo were determined according to TAPPI test method T203 om-09.

The brightness of pulp was measured according to the TAPPI T452 om-08. The viscosity of all samples was measured following TAPPI T230 om-04 using cupriethylenediamine (CED) solution as solvent. The reactivity of samples was determined based on the method reported by Östberg et al. [37].

The solid content of the liquors was determined by vacuum drying at 45 °C for 48 h. The content of soluble silica in the liquors was measured using a UV–Visible spectrophotometer [38]. For the determination of acetic acid, furfural, lignin and carbohydrates content of the liquors, the samples were autoclaved with 4% (w/w) H_2_SO_4_ for 60 min. After autoclaving, the analysis was continued with HPLC as described for the analysis of the solid samples [34]. Lignin content of the black liquors was determined gravimetrically by acid precipitation and centrifugation [39]. The chemical composition such as glucose, and xylose was analyzed by HPLC. Cell growth of the strain was analyzed by measuring the optical density at 600 nm with a UV–Visible spectrophotometer. The total phenolic content in the hydrolysate was determined by a Folin–Ciocalteu assay using gallic acid as a standard. Ethanol concentrations were determined by a gas chromatography (Shimadzu GC-14C, Japan) equipped with a flame ionization detector. A 0.125-cm I.D., 2 m, SS column was used with nitrogen gas (N_2_) as a carrier gas and hydrogen gas (H_2_) as a flaming gas. The injector temperature was 80 °C, and the detector temperature was 220 °C. All measurements were run at least in triplicate.

## Results and discussion

### Effect of alkaline pretreatment on the chemical composition of bamboo chips

During alkaline pretreatment of lignocellulosic biomass, the commonly investigated alkali reagents are NaOH, potassium hydroxide (KOH), lime (Ca(OH)_2_), and ammonia (NH_4_OH). Based on our earlier study on alkaline pretreatment of bamboo chips [15], alkaline pretreatment experiments were carried out with an alkali (OH^−^) charge of 3.0 mmol/g of o.d. bamboo biomass. The alkali loading was expressed in the unit (mmol/g o.d. bamboo biomass) to maintain a stoichiometric ratio of the alkali loading to bamboo mass among different alkali reagents. The temperature and time of the reaction were fixed at 95 °C and 150 min, respectively.

Table 2 summarizes the biomass yield and chemical composition of bamboo following pretreatment with different alkali reagents. Hydrolysis under alkaline conditions causes the cleavage of lignin bonds and glycosidic bonds of hemicellulose as well as the disruption of ester bonds crosslinking lignin and hemicellulose, resulting in the dissolution of hemicellulose and lignin [40]. As shown, the treatment with strong alkali reagents, NaOH (87.2% w/w) and KOH (86.4% w/w), had lower chip yield compared to that of weak alkali reagents, Ca(OH)_2_ (90.4% w/w) and NH_4_OH (90.9% w/w). Based on the chip yield and chemical composition analysis, the residual portion of each bamboo component could be calculated. Lime had the lowest silica removal compared to other alkali reagents (Table 2). After alkaline treatment, about 95% of silica was retained in the treated chips. In contrast, the treatment with NaOH and KOH removed about 99% of original silica from bamboo chips. This is because polyvalent cations, Ca^2+^, interacted with silica and caused co-precipitation by forming insoluble deposits [13]. The removal of silica during ammonia treatment was also very low, only 32.3% of initial silica was removed (Table 2). The removal of 99% of silica from bamboo chips significantly alleviates the silica challenges. The treated substrate is very suitable for the production of high-grade dissolving pulp and bioethanol [41, 42].

**Table 2:** Chemical composition of bamboo chips after alkaline pretreatment

Alkali	Solid yield (%)	Composition (%)			
		Silica	Cellulose	Hemicellulose	Lignin
Untreated	NA	1.12 ± 0.01	47.3 ± 0.32	21.8 ± 0.27	25.3 ± 0.63
NaOH	87.2 ± 0.6	0.012 ± 0.01	51.86 ± 0.36	17.60 ± 0.44	27.97 ± 0.56
KOH	86.1 ± 1.9	0.013 ± 0.01	52.24 ± 0.41	18.03 ± 0.28	28.03 ± 0.78
Ca(OH)_2_	90.4 ± 1.6	1.14 ± 0.03	50.80 ± 0.35	20.67 ± 0.46	27.09 ± 0.49
NH_4_OH	90.9 ± 1.7	0.83 ± 0.04	50.42 ± 0.54	20.82 ± 0.39	27.22 ± 0.71

Treatments were carried out at 90 °C with alkali loading of 3.0 mmol/g o.d. bamboo biomass

All data are shown as mean ± SD

*NA* not applicable, *NaOH* sodium hydroxide, *KOH* potassium hydroxide, *Ca(OH)*_*2*_ calcium hydroxide, *NH*_*4*_ *OH* ammonia

Alkali pretreatment under conditions studied did not show a significant effect on the cellulose and lignin contents (Table 2). More than 95% of cellulose in bamboo was preserved following alkaline pretreatment, which could be attributed to the high crystallinity of cellulose which makes it very recalcitrant towards degradation by alkali [42, 43]. The extracted lignin was determined to be 2.1–5.4% of the initial lignin mass for the four alkali reagents used in alkaline pretreatment (Table 2). The standard error for lignin mass fraction loss was determined to be 1.1–1.8% (out of three runs). The removed lignin could be mainly mono- or oligo-lignols under the studied temperature (95 °C) [44]. Compared with NaOH, more lignin was degraded using KOH; 3.6% (based on the initial lignin) for NaOH versus 5.4% (based on the initial lignin) for KOH. The low lignin removal not only results in the separation of high-purity silica from the APEL by avoiding lignin co-precipitation but also preserves the lignin amount in black liquor obtained from the pulping process.

With regard to the removal of xylan, it can be observed that compared to the weak alkali reagents (Ca(OH)_2_ and NH_4_OH), more xylan was removed when bamboo was treated with the two strong alkali reagents (NaOH and KOH) (Table 2). The treatment with NaOH resulted in the highest xylan removal, which was 29.6% (based on initial xylan), while the xylan removal by the other bases was 28.8, 14.3 and 13.2% (based on initial xylan) for KOH, Ca(OH)_2_, and NH_4_OH, respectively (Table 2). These results are in agreement with those for sugarcane bagasse and sweet sorghum bagasse treated with different alkali reagents [45, 46]. Higher temperature or longer treatment time might be required for lime and ammonia to achieve desirable hemicellulose removal levels. The extraction of about 30% of hemicellulose from bamboo chips should increase the accessibility of residual lignin and carbohydrates to chemicals used in subsequent pulping process, thereby allowing a lower chemical charge to be used [47].

Among the alkali reagents investigated for the removal of silica and hemicellulose from bamboo chips, NaOH is considered to be a better choice due to lower price and less corrosion to the digester than KOH. Moreover, in a typical kraft pulping mill, NaOH is readily available in white liquor. Thus, treated chips and spent liquor obtained from NaOH pretreatment were used for the following experiments.

### Kraft pulping of treated bamboo chips

For the production of dissolving pulp, a pulping stage that fractionates cellulose from lignin and hemicellulose needs to be conducted. A series of experiments on kraft cooking (using NaOH and Na_2_S as cooking chemicals) of the NaOH pretreated chips was carried out.

**Fig. 1:**
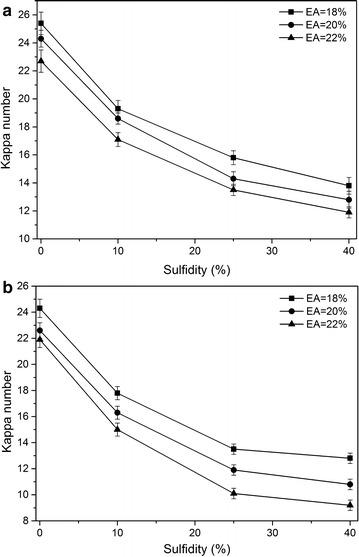
Effect of EA and sulfidity on the kappa number bamboo kraft pulps. **a** 160 °C; **b** 170 °C

During kraft pulping, the degree of delignification is considered one of the most important parameters that reflects the cooking effectiveness. Kappa number, representing residual lignin content in the kraft pulp, was determined and used to evaluate the cooking efficiency. The effects of EA and sulfidity on kappa number of the obtained pulps are shown in Fig. 1. As shown, either increase in EA at a constant sulfidity or increase in sulfidity at a constant EA resulted in an obvious decrease of the kappa number, which is in agreement with previous studies on kraft pulping of bamboo chips [7]. Increasing either the sulfidity or the cooking temperature can result in lower kappa number (Fig. 1). Moreover, results in Fig. 1 further indicated that the effect of sulfidity on kappa number was significant from 10 to 25%; thereafter, the decrease in kappa number started to level off.

To further evaluate the effects of kraft cooking conditions on pulp properties, the chemical composition of obtained kraft pulps was measured (Table 3). The pulp yield was decreased with increasing cooking intensity (higher temperature, higher sulfidity, and higher EA charge) (Table 3). The most important observation is that unbleached kraft pulp with cellulose around 90% (based on o.d. pulp) and hemicellulose less than 10% (based on o.d. pulp) was successfully produced from the alkaline pre-extracted bamboo chips. Under the same conditions, the contents of cellulose and hemicellulose of the kraft pulp from non-extracted bamboo chips were 82 and 16%, respectively. This might be due to the fact that the extraction of lignin and hemicellulose during alkaline pretreatment improved the accessibility bamboo chips to cooking chemicals, resulting in faster removal of lignin and hemicellulose during kraft cooking [26, 48]. The lignin content of the kraft pulp could be decreased to 1.38% (w/w), corresponding 97.8% removal of the original lignin using the combined alkaline pretreatment and kraft pulping. The low residual lignin content in the kraft pulp is expected to benefit the subsequent bleaching process by reducing the demand for bleaching chemicals.

**Table 3:** Effect of EA and sulfidity on yield, chemical composition of kraft pulps from pretreated bamboo chips

Alkaline-pretreated samples	EA (%)	Sulfidity (%)	Total yield^a,c^ (%)	Rejects^a,c^ (%)	Cellulose^b,c^ (%)	Hemicellulose^b,c^ (%)	Lignin^b,c^ (%)	Ash^b,c^ (%)	Silica in pulp^b,c^ (%)
Cooking at 160 °C	18	0	51.8	0.2	80.26 ± 0.35	14.58 ± 0.14	3.56 ± 0.13	0.14 ± 0.01	≈0.01
		10	50.6	0.1	83.28 ± 0.35	13.94 ± 0.32	2.90 ± 0.12	0.12 ± 0.01	ND
		25	49.3	ND	84.30 ± 0.46	13.85 ± 0.41	2.47 ± 0.08	0.08 ± 0.01	ND
		40	48.6	ND	85.02 ± 0.43	13.42 ± 0.25	2.07 ± 0.11	0.11 ± 0.02	≈0.01
	20	0	51.6	0.2	81.64 ± 0.35	13.92 ± 0.25	3.34 ± 0.11	0.13 ± 0.01	ND
		10	49.5	0.1	84.79 ± 0.38	12.53 ± 0.18	2.78 ± 0.09	0.09 ± 0.01	ND
		25	48.2	ND	86.06 ± 0.49	11.99 ± 0.24	2.14 ± 0.06	0.12 ± 0.02	≈0.01
		40	47.4	ND	86.74 ± 0.67	11.65 ± 0.19	1.93 ± 0.08	0.13 ± 0.02	ND
	22	0	50.3	0.1	83.26 ± 0.36	12.36 ± 0.26	3.26 ± 0.09	0.14 ± 0.35	ND
		10	47.7	ND	86.78 ± 0.54	10.65 ± 0.22	2.57 ± 0.12	0.12 ± 0.01	≈0.01
		25	45.5	ND	88.44 ± 0.53	10.21 ± 0.16	2.03 ± 0.09	0.14 ± 0.01	ND
		40	45.0	ND	89.66 ± 0.26	9.73 ± 0.25	1.83 ± 0.13	0.08 ± 0.01	ND
Cooking at 170 °C	18	0	48.9	0.2	81.73 ± 0.25	15.11 ± 0.32	3.28 ± 0.10	0.12 ± 0.01	ND
		10	46.8	ND	84.05 ± 0.36	13.87 ± 0.24	2.67 ± 0.06	0.11 ± 0.01	ND
		25	45.6	ND	84.78 ± 0.32	13.23 ± 0.14	2.13 ± 0.11	0.09 ± 0.01	≈0.01
		40	44.8	ND	85.63 ± 0.44	12.86 ± 0.17	1.98 ± 0.08	0.12 ± 0.01	ND
	20	0	47.8	0.1	83.29 ± 0.45	13.26 ± 0.23	3.19 ± 0.11	0.13 ± 0.02	≈0.01
		10	45.0	ND	86.18 ± 0.22	11.78 ± 0.20	2.45 ± 0.13	0.08 ± 0.01	ND
		25	42.3	ND	87.04 ± 0.49	10.99 ± 0.13	1.65 ± 0.06	0.14 ± 0.02	≈0.01
		40	41.6	ND	87.75 ± 0.19	10.72 ± 0.26	1.52 ± 0.06	0.09 ± 0.01	ND
	22	0	46.4	ND	84.52 ± 0.45	11.13 ± 0.25	3.06 ± 0.12	0.14 ± 0.01	≈0.01
		10	42.7	ND	88.08 ± 0.38	9.67 ± 0.23	2.25 ± 0.08	0.11 ± 0.02	ND
		25	40.2	ND	89.13 ± 0.26	9.04 ± 0.25	1.50 ± 0.05	0.09 ± 0.02	ND
		40	39.1	ND	89.82 ± 0.21	8.76 ± 0.22	1.38 ± 0.07	0.09 ± 0.01	ND

All the data are shown as mean ± SD

*ND* not detected

^a^Calculations were based on original oven-dried chip mass

^b^Calculations were based on oven-dried pretreatment-kraft pulp mass

^c^Values are expressed as averages of two replicate experiments

After sequential steps of NaOH pretreatment and kraft cooking, up to 28% cellulose (based on initial o.d. cellulose mass) was extracted from original bamboo chips (Table 3). With increasing cooking temperature and EA charge, the loss of cellulose increased (Table 3). The peeling reaction and alkaline hydrolysis degrade cellulose into monomers or low molecular weight oligomers, which in turn are transferred into the bulk liquor [41, 49]. In addition, at kraft cooking temperature of 170 °C, both using higher EA charge at lower sulfidity (22% EA and 25% sulfidity) or lower EA charge at higher sulfidity (20% EA and 40% sulfidity) could delignify treated bamboo chips to a residual lignin content of about 1.5% (based on o.d. pulp) (Table 3). However, the utilization of higher EA resulted in lower pulp yield compared to that of higher sulfidity (41.8 versus 40.4%). This could be attributed to the fact that the degradation of carbohydrates is mainly dependent on the concentrations of hydroxide ions in the solution, while Na_2_S accelerates the delignification [49, 50].

In dissolving pulp, the ash is considered a contaminant for the preparation of cellulose derivatives [51, 52]. With alkaline pretreatment, the ash content of the obtained kraft pulp was 0.08–0.14% (on the o.d. pulp), which was below the recommended limits of dissolving grade pulp [51]. The silica content of the pulps was very low (≤0.02% wt/wt), and even undetected in some samples. This low amount of ash content of the dissolving pulp makes it a suitable candidate for the production of high-value cellulosic products such as cellulose acetate, cellulose nitrate, and carboxymethyl cellulose (CMC) [51–53].

### Xylanase treatment and cold caustic extraction (CCE) of kraft pulp

For the production of dissolving grade pulp, cellulose yield at a low level of residual hemicellulose and lignin content is a critical parameter at an industrial scale [54]. Thus, operations that could extract hemicellulose and lignin while minimizing cellulose degradation are required after kraft pulping. The kraft pulp from one representative kraft pulping run with about 90% cellulose and 9% hemicellulose was selected for subsequent study on hemicellulose removal. The kraft pulping conditions were 22% EA and 25% sulfidity at 170 °C.

**Fig. 2:**
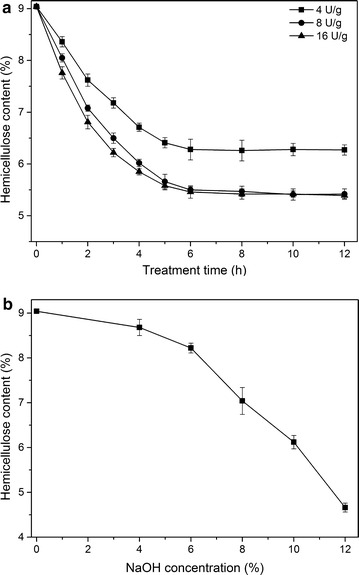
**C**omparison of hemicellulose content of pulps from different treatments. **a** Xylanase treatment at temperature 60 °C, pH 6, and 10% pulp consistency; **b** CCE at temperature 30 °C and 10% pulp consistency for 45 min

The effect of two treatment methods, xylanase and CCE, on the residual hemicellulose content is shown in Fig. 2. The residual hemicellulose content was about 6.28% (w/w) after 6-h treatment using a xylanase dosage of 4 U/g o.d. pulp (Fig. 2a). With increasing xylanase dosage to 8 U/g or even 16 U/g o.d. pulp, time course analysis demonstrated that the residual hemicellulose content of the pulp was reduced to 5.49% (w/w) after 6-h treatment. With prolonged treatment up to 12 h, the hemicellulose content of the pulp only decreased only slightly. For example, with a xylanase charge of 8 U/g o.d. pulp, after 12-h treatment, the hemicellulose content in the pulp was 5.41% (w/w), representing only 1.5% hemicellulose removal beyond the 6-h treatment. This could be due to molecular interactions between hemicellulose and the lignocellulosic matrix which cannot be hydrolyzed by the used enzyme system (xylanase).

For the removal of hemicellulose with CCE (Fig. 2b), the lower NaOH concentration (4 and 6%) affected hemicellulose content slightly, which is in accordance with previous studies using softwood and hardwood pulps [27, 55, 56]. With increasing the NaOH concentration over 8%, a significant decrease of hemicellulose content was observed. The use of high NaOH concentration during CCE results in more swelling of cellulosic fibers, thereby opening up the fiber structure and increasing the internal surface area, which improves the accessibility of hemicellulose to NaOH and facilitates the diffusion of hemicellulose degradation products [41, 57, 58]. By treating the pulp with CCE at 12% NaOH concentration, the residual hemicellulose content of pulp was 4.66% (w/w). These values are lower than those reported by Gehmayr et al. [55] who treated pre-hydrolysis kraft Eucalyptus pulp with 10% (4.2% w/w) NaOH. This distinction can be explained by the differences of the chemical composition and the fiber structure between the two pulps and the NaOH concentrations used. Comparing the residual hemicellulose content of pulps obtained from the two treatment methods, cold caustic extraction of the pulp at 12% NaOH concentration removed more hemicellulose than xylanase treatment (Fig. 2). However, the use of high alkali concentration in CCE causes several complications in the industrial processes and negatively affects the quality of the final pulp. It has been reported that using alkali concentration higher than 8% for CCE creates problems during washing/purification process with water due to the strongly swollen pulp fibers [41, 56]. The utilization of high NaOH concentration also changes cellulose I (native cellulose) to cellulose II (regenerated cellulose), which decreases the reactivity of the final pulp [59, 60]; this should be minimized during the production of high-grade dissolving pulp. In addition, the residual hemicellulose content of the treated pulps, 5.49% (w/w) and 7.14% (w/w) for xylanase treatment and CCE (at 8% NaOH concentration), respectively, were still very high for high-grade dissolving pulp. Therefore, the effect of xylanase treatment of pulp prior to CCE on hemicellulose removal was investigated to reduce the consumption of enzyme and NaOH and to extract more hemicellulose.

**Fig. 3:**
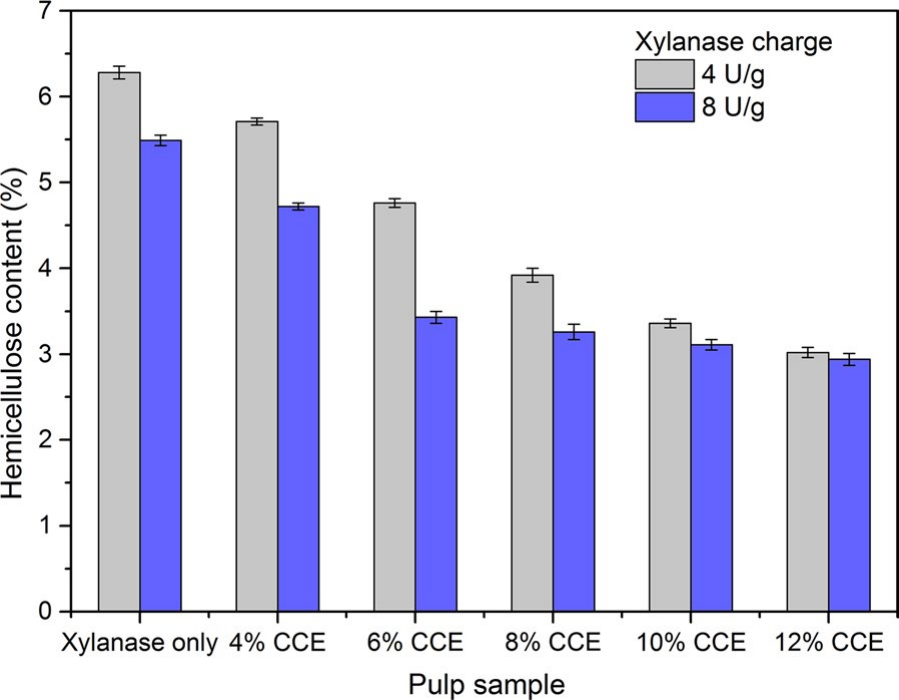
Hemicellulose content of pulps from the sequential treatment process of xylanase and CCE. Xylanase treatment: temperature 60 °C, pH 6, pulp consistency 10%; CCE process: temperature 30 °C, time 45 min, pulp consistency 10%. 4–12% means the used NaOH concentration during CCE process

The 6-h enzyme-treated pulp samples with xylanase charges of 4 and 8 U/g were subjected to CCE. Figure 3 shows the effect of sequential treatments with xylanase and CCE on the residual hemicellulose content of pulp samples. As shown, the combination of treatment methods significantly reduced the residual hemicellulose content in the pulp. The xylanase pretreatment also improved hemicellulose removal during CCE (even at low NaOH concentration). For example, using 4% NaOH to extract hemicellulose from pulp obtained after 8 U/g xylanase treatment, the hemicellulose content reduced from 5.49 to 4.72% (w/w), which was comparable to that of single stage of CCE (4.66%) with 12% NaOH. This could be attributed to the fact that xylanase treatment increased the pore volume of fiber, thereby increasing the accessibility of xylan to NaOH during the subsequent CCE process [30].

Moreover, when using lower NaOH concentration (4–8%) during CCE, the difference of the residual hemicellulose content between the two xylanase-treated pulps was larger (Fig. 3). For example, with 6% NaOH treatment, the hemicellulose content of 8 U/g xylanase-treated pulp was 3.43 versus 4.76% (w/w) for 4 U/g xylanase-treated pulp. One likely reason might be pulps contained different hemicellulose contents after xylanase treatment. With the increase of the NaOH concentration (≥8%) in the CCE stage, similar hemicellulose content was obtained from the two xylanase-treated pulps (Fig. 3). A reasonable explanation could be as the xylan content drops, the residual xylan might be tightly associated with the pulp making it difficult to remove.

### Bleaching of xylanase-CCE-treated pulp

To evaluate whether the proposed technology can produce high-quality dissolving pulp, the pulp samples obtained by sequential xylanase (4 and 8 U/g)-CCE (at 4, 6 and 8% NaOH) treatment were subjected to the elemental chlorine-free (ECF) bleaching sequence, D-E-D. As shown in Table 4, with the proposed procedure of alkaline pretreatment-kraft cooking-xylanase treatment-CCE-bleaching (D-E-D), the obtained pulp essentially met the requirements of an acetate grade pulp with an α-cellulose content higher than 97%, hemicellulose content lower than 2%, ash content lower than 0.08%, and intrinsic viscosity between 6.24 and 6.64 mPa s [53, 61]. Moreover, the final pulp yield of 30–33% (based on initial o.d. chip mass) is also in the acceptable range for the production of dissolving pulp from lignocellulosic feedstocks [51, 62].

**Table 4:** Yield, hemicellulose content, α-cellulose, ash content, brightness, reactivity, and viscosity of bleached (D-E-D) bamboo dissolving pulp

Sample	Yield^a^ (%)	Hemicellulose^b^ (%)	α-cellulose^b^ (%)	Ash^b^ (%)	Brightness (% ISO)	Viscosity (mPa s)	Fock reactivity (%)
Control	36.2	7.49 ± 0.12	92.06 ± 0.03	0.09 ± 0.01	85.6 ± 0.1	6.94 ± 0.04	22.3 ± 0.78
Xylanase^c^ and 4% CCE	33.8	3.87 ± 0.11	95.72 ± 0.06	0.08 ± 0.02	86.2 ± 0.1	6.45 ± 0.02	32.6 ± 0.24
Xylanase^c^ and 6% CCE	32.6	2.45 ± 0.06	97.18 ± 0.13	0.06 ± 0.01	86.7 ± 0.1	6.39 ± 0.02	43.4 ± 0.45
Xylanase^c^ and 8% CCE	31.4	1.58 ± 0.10	97.82 ± 0.10	0.07 ± 0.02	87.6 ± 0.1	6.24 ± 0.01	23.4 ± 0.58
Xylanase^d^ and 4% CCE	32.0	2.67 ± 0.08	96.98 ± 0.15	0.05 ± 0.01	86.9 ± 0.1	6.38 ± 0.02	34.6 ± 0.62
Xylanase^d^ and 6% CCE	31.8	1.36 ± 0.05	97.84 ± 0.11	0.08 ± 0.02	87.8 ± 0.1	6.25 ± 0.01	44.7 ± 0.25
Xylanase^d^ and 8% CCE	30.2	1.14 ± 0.07	98.06 ± 0.05	0.06 ± 0.01	88.3 ± 0.1	6.12 ± 0.02	22.1 ± 0.93

4–8% CCE means the NaOH concentration used for cold caustic extraction

*Control* the pulp was treated as the same operations used for xylanase treatment without using xylanase and NaOH

^a^Calculations were based on original oven-dried chip mass

^b^Calculations were based on oven-dried corresponding pulp mass

^c^Pulp was treated with xylanase 4 U/g o.d. pulp at 60 °C and pH 6 for 6 h

^d^Pulp was treated with xylanase 8 U/g o.d. pulp at 60 °C and pH 6 for 6 h

Compared to the control, the utilization of the sequential treatment (xylanase and CCE) also improved the Fock’s reactivity of the bamboo dissolving pulp. The pulp reactivity, measured with xanthation chemicals used in the viscose process, increased from 22.3 to 44.7% (Table 4). However, when high NaOH concentrations were used during CCE, the pulp Fock’s reactivity decreased. For example, with the 8 U/g xylanase-treated pulp, the reactivity decreased from 44.7% (at 6% NaOH concentration) to 22.1% (at 8% NaOH concentration). This might be caused by the formation of cellulose II during CCE process [27]. Intrinsic viscosity of dissolving pulp represents the degree of polymerization of cellulose. Pulp samples obtained from 4 U/g xylanase treatment generally had higher viscosity than 8 U/g xylanase-treated pulps, but both were lower than the control. A possible reason might be the xylanase treatment causes slightly degradation of cellulose. Pulp brightness was also improved by the combination treatment (Table 4), which means the combination of xylanase treatment and CCE improved the delignification during the bleaching process. This might be due to the removal of more hemicellulose with xylanase-CCE treatment, which improves the accessibility of lignin to bleaching chemicals [55]. Moreover, the charges of bleaching chemicals used in this study were lower than that in the previous studies [2, 52], indicating a lower requirement of chemicals for the bleaching stage can be achieved.

Based on the proposed scheme, high-quality dissolving pulp (>97% α-cellulose, <2% hemicellulose and <0.08% ash) was produced from bamboo. Thus, the combination of alkaline pretreatment, alkaline pulping, xylanase treatment, CCE, and bleaching technologies is a promising approach, and bamboo is a promising alternative feedstock, for the production of high-grade dissolving pulp.

### Recovery of dissolved materials from the spent liquors

The dissolved compounds detected in the alkaline extraction liquor included inorganic chemicals (NaOH and Na_2_CO_3_), sugars, lignin, sodium acetate, sodium silicate, and lignin-derived compounds. For economical and eco-friendly production of pulp and fitting the pulping process well into the integrated forest biorefinery concept, the dissolved products should be recovered for conversion into “green chemicals” and fuels. Table 5 summarizes the chemical composition of the two spent liquors before and after the recovery of silica, lignin, and carbohydrates. Alkaline pretreatment was carried out with commercial bamboo chips with NaOH concentration of 3 mmol/g at 95 °C for 150 min with a liquid-to-wood ratio of 4 L/kg in the laboratory. The silica content of alkaline pre-extraction liquor (APEL) was 4.56% of the total solid. Based on the solid content and liquid-to-wood ratio used for the extraction, it was calculated that about 99% of silica in raw chips was removed. This was confirmed by the measurement of silica content of NaOH-extracted chips (≈0.013% w/w). As expected, the lignin content of the APEL was low at 3.77% of the total solid; this low concentration is favorable for silica recovery and maintenance of lignin concentration in the black liquor. Sugars, xylan, glucan, galactan, and arabinan, comprised 33.37% of the total solid in the liquor (APEL). The components, silica and sugars, must be removed prior to sending the liquor to the kraft recovery circle where recovery of the alkali used in the extraction will occur [41].

**Table 5:** Recovery of silica, lignin, and carbohydrates from the two liquors of bamboo processing

Sample	Solid content (%)	Chemical composition (%)^a^								
		Ash (without silica)	Silica	Glucan	Xylan	Galactan	Arabinan	Lignin	Phenolic	Others
APEL	5.81 ± 0.06	47.71 ± 0.12	4.56 ± 0.10	7.47 ± 0.04	24.14 ± 0.23	0.82 ± 0.03	0.94 ± 0.23	3.77 ± 0.16	≈0.13	10.28 ± 0.11
BL	17.36 ± 0.04	34.32 ± 0.14	ND	12.85 ± 0.48	15.48 ± 0.47	0.46 ± 0.05	0.58 ± 0.04	30.22 ± 0.36	3.75 ± 0.11	3.23 ± 0.13

Recovery	Silica (%)^b^	Lignin (%)^b^	Carbohydrates (%)^b^	Composition of carbohydrates (%)						
				Glucan	Xylan	Galactan	Arabinan	Lignin	Phenolic	Others
APEL	1.06 ± 0.04	NA	10.13 ± 0.10	16.26 ± 0.04	53.44 ± 0.23	1.54 ± 0.03	2.21 ± 0.23	7.40 ± 0.16	≈0.13	16.64 ± 0.11
BL	NA	19.74 ± 0.14	21.06 ± 0.23	39.12 ± 0.06	48.29 ± 0.15	0.90 ± 0.11	1.12 ± 0.16	0.78 ± 0.17	5.54 ± 0.16	3.06 ± 0.14

All data are shown as mean ± SD

*ND* not detected, *NA* not applicable, *APEL* alkaline pre-extraction liquor, *BL* black liquor, *others* includes acetyl and uronic acids groups, extractives

^a^Values were expressed as the percentage of the total dry solids

^b^Calculations were based on original oven-dried chip mass

The black liquor obtained from kraft pulping of extracted chips had a solid content of 17.36%. Silica was not detected in the black liquor, which greatly enhanced the potential of producing high-purity lignin from the black liquor for value-added products. The content of sugars in the black liquor was determined to be 29.37% of the solid content. The lignin content in the black liquor was 30.22% of the total solid. The ash (without silica) in the black liquor consisted of mainly sodium compounds, which can be recovered, after the recovery of lignin and sugars, through the chemical recovery circle [41].

Silica was separated from the APEL by reducing the pH of the liquor to pH 8 with CO_2_ (flue gas could be used in pulp mills). About 1.06% (based on original o.d. bamboo biomass) corresponding to 94.64% of original silica was recovered (Table 5). The recovered high-purity silica, mainly of amorphous structure, can be used for the production of thixotropic agents, pharmaceuticals, film substrates, electric and thermal insulators, composite fillers, etc. [16]. The CO_2_-treated liquor was subjected to nanomembrane filtration to concentrate hemicellulosic sugars (filtration was conducted at 500 psi for 150 min). The sugar recovery was 10.13% (dry weight) of original o.d. bamboo biomass. After separating silica from the APEL, results demonstrated that the precipitate of CO_2_-treated APEL contained 73.55% of sugars, in which contents of hemicellulosic sugars (xylan, galactan, and arabinan) and glucan were 57.19 and 16.36%, respectively (Table 5). The determined contents of phenolics, lignin, and “others” were 0.13, 7.40 and 16.64%, respectively (Table 5).

Lignin was separated from the black liquor by lowering the pH to 2–3 with H_2_SO_4_. The lignin yield was about 19.74% (based on original o.d. chip mass), which corresponded to 78.05% of the starting lignin in original bamboo chips (Table 5). The approximately 22% lignin loss might be due to unrecovered lignin dissolved in the APEL, residual lignin in kraft pulp, wash-off of lignin precipitate, and lignin degradation during pulping. Since silica was recovered prior to lignin precipitation, the ash content of the separated lignin was only 0.71% (w/w), indicating that the recovered lignin can be a potential source for high-value polymeric materials development [31, 63].

After the separation of lignin, the dissolved sugars (derived from hemicellulose and cellulose) in the black liquor were concentrated using nanomembrane filtration (filtration was conducted at 500 psi for 150 min). The obtained concentrate contained 89.43% of sugars, in which the contents of hemicellulosic sugars (xylan, galactan, and arabinan) and glucan were 50.31 and 39.12%, respectively (Table 5). The measured contents of phenolic compounds and “others” were 5.54 and 3.06%, respectively. According to above results, recovered concentrates from both APEL and black liquor contain high levels of sugar. Thus, the two concentrates were mixed and fermented to produce ethanol.

### Overliming-detoxification and enzymatic hydrolysis of recovered sugars

To remove undesired components from the recovered sugar concentrates, overliming, which has been widely used for the removal of phenolic inhibitors from hydrolysate [64, 65], was used to make fermentable hydrolysates. Table 6 shows the chemical composition of the sugar mixture after overliming. The yield of overliming was 81.95% (based on the initial recovered sugar mixture), resulting in a sugar yield of about 25.56% of initial o.d. bamboo biomass. In the detoxified sugar syrup, the contents of hemicellulose- and cellulose-derived sugars and oligomeric sugars were 42.33 and 27.19 g/L, respectively, while acetic acid and furfural were not detected with the methodology used (Table 6). This solution with high sugar concentrations and low levels of fermentation inhibitors and catalyst poisons is very attractive for downstream processing to produce fuels and value-added products.

**Table 6:** Chemical composition of the sugar mixture before and after enzymatic hydrolysis

Enzyme loading	Composition (g/L)						
	Xylose	Glucose	Arabinose	Galactose	Gluco-oligomers	Xylo-oligomers	Furfural
Overliming–detoxification	15.74 ± 0.26	10.32 ± 0.23	0.37 ± 0.05	0.51 ± 0.04	25.69 ± 0.45	16.87 ± 0.65	0
1 mg/g	39.72 ± 0.34	26.13 ± 0.14	0.36 ± 0.07	0.53 ± 0.11	1.62 ± 0.14	1.05 ± 0.05	0
3 mg/g	39.81 ± 0.41	26.22 ± 0.15	0.38 ± 0.11	0.52 ± 0.15	1.48 ± 0.23	0.92 ± 0.08	0
10 mg/g	40.01 ± 0.35	26.38 ± 0.11	0.37 ± 0.06	0.49 ± 0.09	1.39 ± 0.14	0.76 ± 0.09	0

All data are shown as mean ± SD

The detoxified sugar syrup was subjected to enzymatic hydrolysis with different enzyme loadings. Table 6 also summarizes the results of enzymatic hydrolysis of recovered sugars and the concentrations of generated monomeric and oligomeric sugars. At enzyme loading of 1 mg protein/g sugar, the hydrolysate contained 65.85 g/L monomeric sugars with 39.72 g/L xylose and 26.13 g/L glucose. The concentration of oligomeric glucose and xylose was 2.67 g/L. Furfural and acetic acid, inhibitors of fermentation, were not detected in the sugar hydrolysate. With the increase of enzyme loading to 10 mg protein/g sugar, no significant increase in the amount of monomeric sugar concentration was observed. Thus, enzyme loading of 1 mg protein/g sugar was selected for the enzymatic hydrolysis. The generated enzymatic hydrolysate with high sugar concentration and low levels of inhibitors can be used for fermentation to produce biofuels.

### Fermentation of enzymatic hydrolysate

**Fig. 4:**
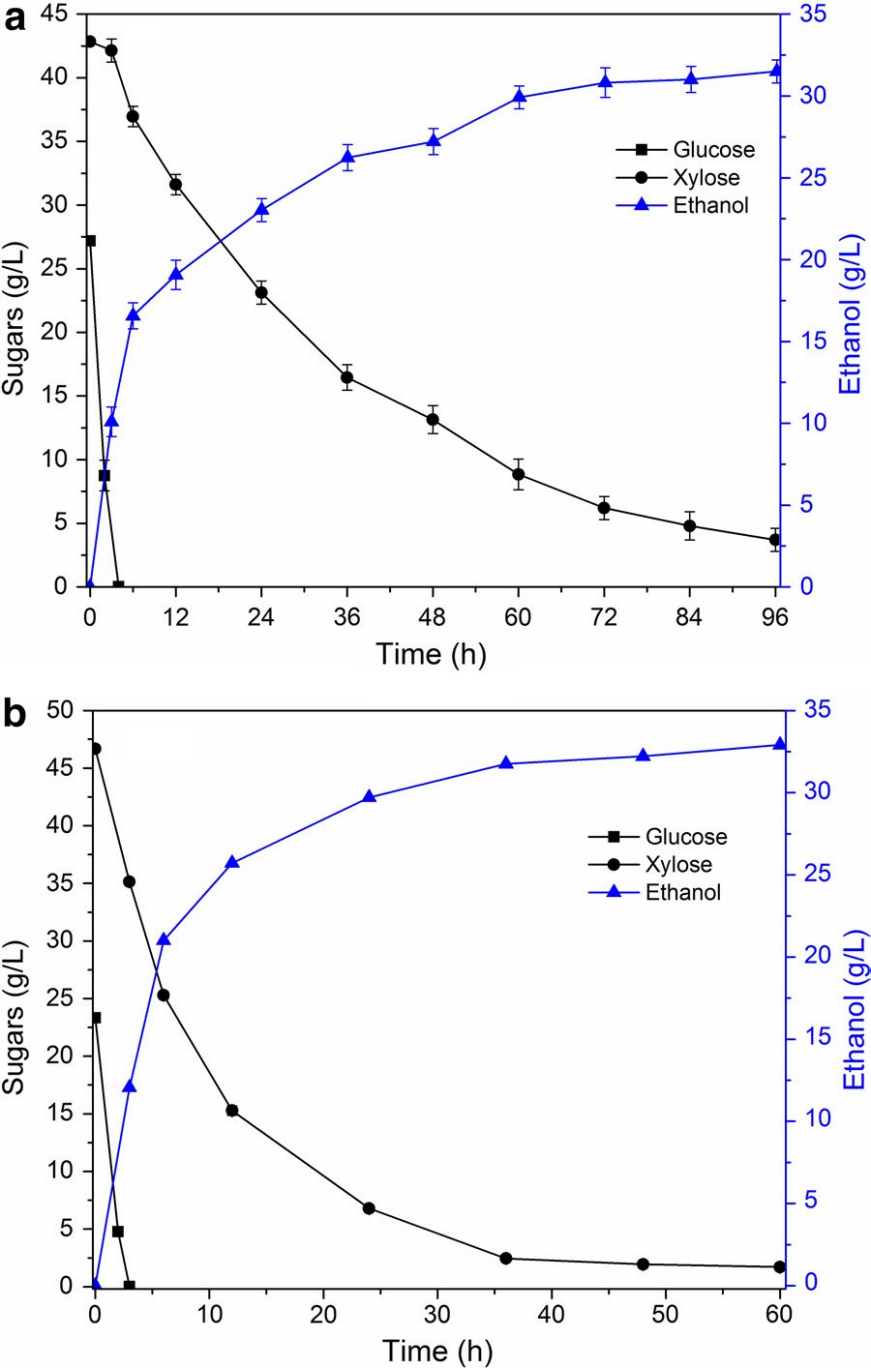
Fermentation performance of using the Saccharomyces cerevisiae BFIS strain. **a** Recovered sugar hydrolysate from bamboo processing; **b** a standard sugar mixture. Fermentation was initiated with a high cell density (initial strain cell = 1.0 g/L) at 30 °C, pH 5.0, and 150 rpm under anaerobic conditions. Each experiment was performed in triplicate, and the *error bars* indicate the standard deviation of the mean of triplicate values

Following enzymatic hydrolysis, the generated hydrolysate was fermented by the *S. cerevisiae* BSIF strain developed for the production of ethanol. In addition, to evaluate the effect of inhibitors present in the sugar hydrolysate on the fermentation efficiency, the testing of a mixture of standard synthetic sugars (glucose and xylose) was also conducted in parallel. Figure 4 presents the fermentation process for the studied sugar hydrolysate and standard synthetic sugar with the studied strain. It can be observed that the glucose was fermented much faster than xylose (Fig. 4). For example, glucose in the studied hydrolysate was readily fermented with BSIF strain within 4 h, while only approximately 80% of xylose was consumed in 48 h (Fig. 4a). The maximum consumption rate of glucose (6.31 g/L/h in 2 h) in the bamboo hydrolysate was 68% of the control (9.29 g/L/h in 2 h). The xylose consumption in 24 h was 0.89 g/L/h, which was 54% of that of the control (1.66 g/L/h in 24 h) (Fig. 4). The observed slow fermentation rates of sugars generated from bamboo processing may be due to the presence of small amounts of arabinose and acetic acid [45, 47]. The overall xylose consumption rate was 0.42 g/L/h (0–96 h).

Moreover, as shown in Fig. 4a, in 96-h fermentation, the *S. cerevisiae* BSIF strain resulted in high sugar conversion and ethanol yield, which were 93.2% and 0.46 g/g consumed sugar, respectively. The concentration of obtained ethanol was more than 30 g/L, which can be concentrated to a desired concentration without consuming a great amount of energy. The ethanol yield of 0.46 g/g sugar is equivalent to 90.2% of the maximum theoretical yield. Importantly, the sugar consumption and the ethanol yield of studied sugar hydrolysate (Fig. 4a) were similar to those of the mixture of synthetic sugars (Fig. 4b). Figure 4 also reveals that the high ethanol yield (0.46 g/g sugar consumed) of the bamboo hydrolysate was maintained throughout the 96-h fermentation, indicating the conversion of xylose to ethanol occurred efficiently both in glucose/xylose co-fermentation phase (0–4 h) and the xylose-only fermentation phase (4–96 h). However, compared to glucose, the consumption of xylose was much slower. It is necessary to develop new stains for faster fermentation of xylose to increase the mill efficiency.

### Potential of the proposed technology

**Fig. 5:**
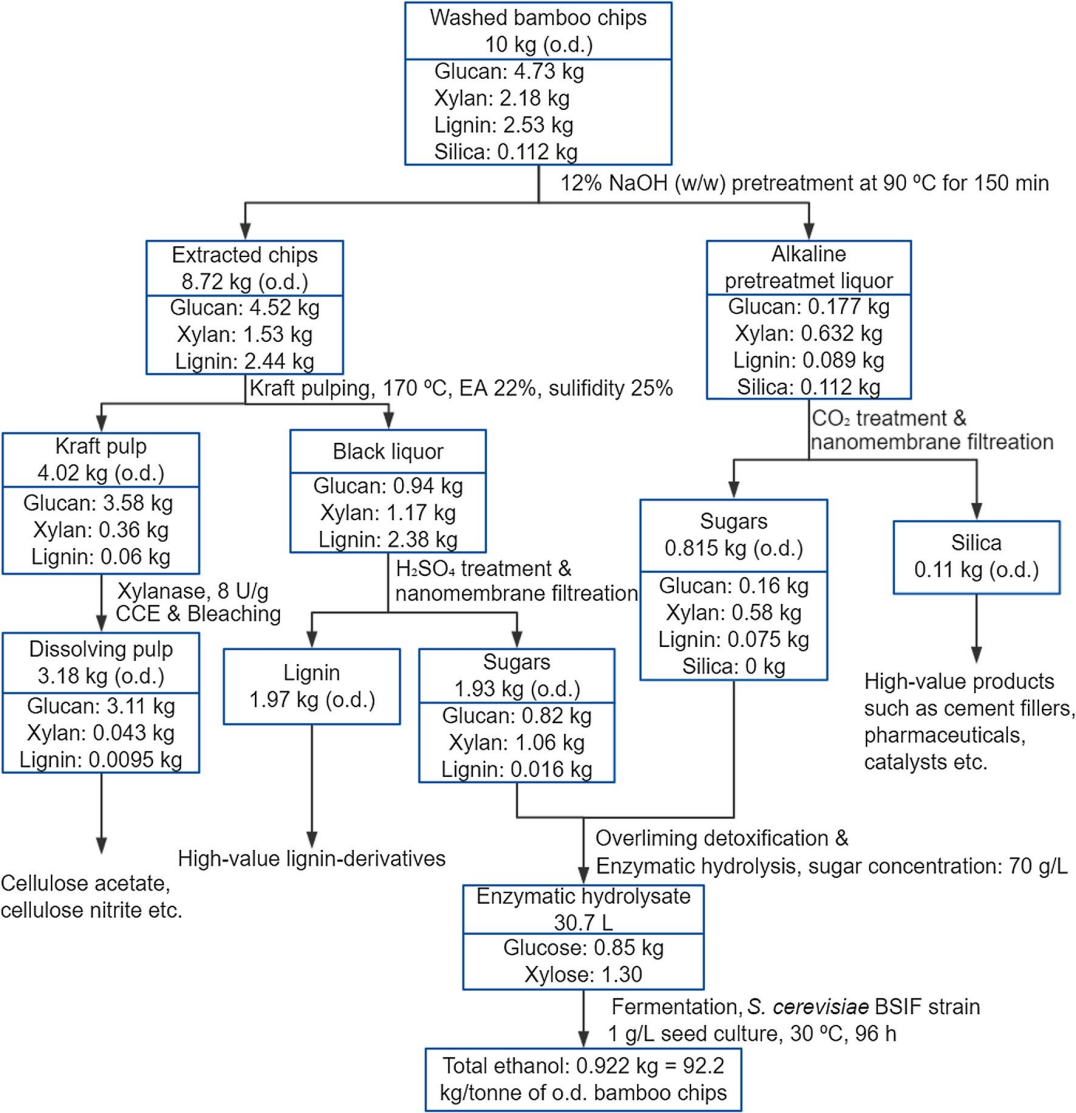
Mass balance for the overall process for the production of dissolving pulp, ethanol, silica, and lignin from bamboo chips. *EA* effective alkali, *CCE* cold caustic extraction, *CO*_*2*_ carbon dioxide, *H*_*2*_ *SO*_*4*_ sulfuric acid

Based on our findings, we propose a process for the transformation of a conventional kraft pulp mill into an integrated forest biorefinery unit. Figure 5 shows the proposed process flow for the production of high-grade dissolving pulp, bioethanol, and high-purity silica and lignin from bamboo chips. In the proposed process, the main chemical used is alkali, which is abundant in white liquor in a typical kraft pulp/dissolving pulp mill. Moreover, the established cooking and recovery equipment of the kraft mill can be used, thus, reducing the total capital investment required. In addition, the utilization of alkaline pretreatment for the production of dissolving grade pulp also reduces the corrosion of digesters caused by acid, which is generated during acidic pre-hydrolysis of lignocellulosic biomasses.

In the proposed scheme, bamboo chips are treated with alkali solution under low temperatures from which a liquor (APEL) rich in silica and hemicellulose is produced. The silica-free substrate serves as raw material for the production of high-grade dissolving pulp or kraft pulp using a conventional kraft pulping process. During kraft pulping, most of lignin, and a proportion of hemicellulose and cellulose are dissolved into the spent liquor, named black liquor. The dissolved materials, including silica, lignin, hemicellulosic sugars, and degraded cellulose, in both the APEL and black liquor are recovered. The recovered silica and lignin are potential starting materials for various high-value applications, while the recovered sugars from the two liquors are fermented for the production of ethanol.

A mass balance starting from 10 kg (o.d.) of green bamboo chips for our overall process is shown in Fig. 5. Based on this mass balance, 311 kg dissolving pulp, 92.2 kg ethanol, 10.6 kg silica, and 197 kg lignin per tonne o.d. bamboo chips were obtained. In the dissolving pulp production step, the generated dissolving pulp had α-cellulose content higher than 97%, hemicellulose content less than 2% and ash content below 0.1%, indicating that it can be used for the production of cellulose acetate and cellulose nitrite [53, 61]. In the ethanol production step, there was 69.0% (calculated based on the theoretical ethanol generation ratio: 0.51 g/g sugar) ethanol recovery based on the total recovered organics before detoxification and enzymatic hydrolysis. This low recovery was due to the high content of phenolic compounds, derived from lignin during kraft pulping, acetic acid, and some extractives. In addition, with regard to the differences between the two sum values of output products before and after ethanol fermentation, during the ethanol fermentation process, the ethanol yield is 0.46 g/g sugar, showing that the total weight of the final output products is smaller than the output products before ethanol fermentation. The overall bamboo biomass recovery (total mass of recovered biomass components) of the proposed process showed 76.59% of initial mass of o.d. biomass, which is a good mass balance for the biorefinery process [66].

Additionally, to enhance the commercial applicability of the process and reduce the utilization of chemicals such as H_2_SO_4_, the dissolved lignin in the kraft black liquor could be separated by ultrafiltration membrane technology [67, 68], during which lignin can be fractionated based on molecular weight. The characterization and utilization of recovered silica and lignin will be further investigated; they will also become excellent sustainable raw material for bio-based products. The methods developed in this study can be utilized with other high silica content biomasses.

## Conclusion

In the present study, an integrated process consisting of production of dissolving pulp, ethanol, high-purity silica, and lignin from bamboo was designed based on the conventional kraft pulping process. All the silica was removed from bamboo chips by treating with 12% NaOH (w/w based o.d. bamboo) at 95 °C for 150 min. Kraft pulp with cellulose content of about 90% (w/w) and hemicellulose content less than 10% (w/w) was produced from the alkali-treated silica-free bamboo chips. After xylanase treatment (8 U/g), cold caustic extraction (at 6% NaOH concentration), and ECF bleaching sequence, dissolving pulp with α-cellulose higher than 97%, hemicellulose lower than 2%, and ash around 0.08% was produced. The pulp is a good starting material for cellulose acetate production.

Moreover, dissolved materials during alkaline pretreatment and kraft pulping were also recovered. With the proposed recovery process, 10.6 kg silica, 197 kg lignin, and 92.2 kg ethanol per tonne bamboo chips (o.d.) were produced in addition to the dissolving pulp. Silica-associated problems in kraft pulping and dissolving pulp production can be greatly alleviated by using the proposed process to process bamboo.

## Abbreviations

NaOH: sodium hydroxide
KOH: potassium hydroxide
Ca(OH)_2_: calcium hydroxide
NH_4_OH: ammonia
H_2_SO_4_: sulfuric acid
Na_2_S: sodium sulfide
Na_2_CO_3_: sodium carbonate
NREL: National Renewable Energy Laboratory
APEL: alkaline pre-extraction liquor
BL: black liquor
*S. **cerevisiae*: *Saccharomyces cerevisiae*
EA: effective alkali
CO_2_: carbon dioxide
CCE: cold caustic extraction
ECF: element chlorine free
D-E-D: chlorine dioxide-sodium hydroxide-chlorine dioxide
HPLC: high performance liquid chromatography
